# A Farm Kid Paradox

**DOI:** 10.1002/nad.12118

**Published:** 2019-11-20

**Authors:** Casper Bendixsen

**Affiliations:** ^1^ Marshfield Clinic Research Institute

**Keywords:** applied anthropology, workplace safety, child labor, immunology, rural culture

## Abstract

Children are commonly and often fatally injured in agricultural settings that include family farms, constituting fully half of all working youth fatalities in the United States. Yet certain aspects of farm life that expose children to harm are also linked to positive health outcomes, a phenomenon that this essay terms “the farm kid paradox.” It reviews applied anthropological research on the differential intertwining of health and hazard, and reflects on the role of the anthropologist as a broker of facts and concepts between diverse stakeholders and farm environments.

Family farms are seats of social–cultural life in rural North America. Farm kids are elemental to their stories, occupying an ambiguous role within the “agrifamily system” (Bennett and Kohl 1982). While most US industries barred children as workers in the early twentieth century, farm kids were not so easily excluded from workplaces that are often also their homes. Children on farms still rise early in the morning to gather eggs, feed calves, and irrigate crops. They also still play on haystacks, swing from ropes, and ride bikes in the yard. Today, almost one million US children live on farms and over half that number report working there; an additional quarter‐million work on farms that are not their residence, from neighbor kids working summer jobs to immigrant children working alongside their parents or simply accompanying them to the fields (CDC 2014). Simply put, most farms (and not just the owner‐operated kinds most commonly glossed as *family farms*) remain places where the domains of work and home intermingle. This has profound effects on the daily lives of children in these settings.

As farm production intensified over the course of the twentieth century, new hazards evolved with the increased use of machinery and application of chemicals (Murphy 1992). Tractors, for instance, are today the leading cause of fatalities for children on farms in the United States, whether as operators, extra riders, or bystanders. Larger farms are subject to increased oversight from regulatory agencies like the Occupational Safety and Health Administration, and most must provide workers’ compensation insurance. But these labor protections, many of which trace their origins to industrial workplaces, do not mesh easily with more limited logics of state intervention into the home (see also Lash 2017 on how these logics play out across race and class). As a result, it is not widely recognized that children are commonly and often fatally injured in agricultural settings that include family farms, constituting fully half of all working youth fatalities in the United States (Perritt et al. 2017).

What is the role of anthropology in confronting this issue? Earlier in my career, I wrote appreciatively about agrarian life and the raising of farm kids as a multigenerational ethical project of kinship, animal husbandry, and land stewardship (Bendixsen 2014). In my current role as an applied anthropologist, though, I work with agricultural communities and health and safety professionals toward the mitigation of farm hazards, including those specific to children. My work at the National Farm Medicine Center over the past six years has challenged me to integrate these perspectives and to confront an empirical phenomenon that I call *the farm kid paradox*: Certain aspects of farm life that expose children to harm are also linked to positive health outcomes. Disentangling the healthful from the hazardous thus proves to be a tall order in a working landscape that resists such tidy distinctions (cf. Roberts 2017).

Today, for instance, biomedical discoveries are validating the belief that farm kids have better immune and respiratory health than their nonfarm counterparts (von Mutius and Vercelli 2010). Seen through an ecosocial lens (Krieger 2011), agrarian ideals of healthy working bodies have arguably been updated for a new, more‐than‐natural context of industrial exposure. Research tells us that exposures to the work environment during pregnancy are where the respiratory health of children begins, and yet we know that this is also a period of vulnerability. On‐farm in utero exposures to pesticides can also lead to significantly increased risks of childhood brain tumors (Kunkle et al. 2014). Meanwhile, the capacity to regulate degrees of exposure for oneself or one’s children is related to social power, whether in terms of race, gender, citizenship, or economic status.

At the National Farm Medicine Center, we seek to contribute to this emerging body of research on the differential intertwining of health and hazard. One of our research teams is investigating the immunological and respiratory health of farm kids from in utero through eight years of age. Through biological samples, surveys, and interviews, we hope to learn what elements and behaviors in farm environments produce decreased allergen sensitization and reduced viral infection in early life (see Ludka‐Gaulke et al. 2018). Our preliminary results suggest that it is likely endotoxins related to large livestock and their feed that help “exercise” a farm kid’s immune system during gestation and in early childhood, resulting in lower rates of allergy and asthma. It also appears that increased time of exposure and diversity of exposures; that is, more kinds of animals and feed have positive effects. These results support the findings of similar studies of Plain community children and their protection from asthma (e.g., Stein et al. 2016). The paradox, however, lies in the fact that large livestock is also the number one cause of nonfatal child injury on farms. Both participation in and separation from farm exposures carry consequences for children’s health that we are still striving to understand.

The coexistence of hazardous working landscapes and the imaginative landscapes of childhood play was further brought home in a recent interview with a mother, who recalled:

Well, we’ve been reading the *Narnia* books recently. So the kids have been imagining the battles and scenes in the barn and in the yard. They are in the barn, around the cows and feed, defending their land. They run and laugh. It’s really quite the production. But all play stops when the skid steer [a quick‐turning piece of farm equipment] is running. The kids know to stay within an arm’s length of a building until they make eye contact with Dad. Then they can cross the yard back to the house. The neighbors ran over one of their kids with a skid steer, so my husband and I live in some fear.

This mom is clear‐eyed about the farm as a place of play among the trappings of agriculture as well as a site of danger where child fatalities are real. A well‐meaning outsider might argue that these intimately learned lessons are not a generalizable model of injury prevention. They rely too much on the child’s behavior, it might be said, rather than separating the child from the skid steer by regulations or technical modification. But if we consider the arm’s‐length rule as a community‐based solution for child runovers, it may well improve outcomes on other family operations. This is despite not being engineered perfectly and not likely being generalizable to settings where the children are less well known to the drivers. In our work, an openness to different mechanisms of intervention is key, especially given the diverse types of farm environments that we encounter across rural America.

Perhaps there are health and safety professionals who will always struggle with the idea of bringing up a child in close proximity to dangerous machinery and animals whose behavior can never be fully predicted. Perhaps farm parents will always struggle with the notion that a good childhood is one that is insulated from those proximities. Without a doubt, the ethical quandaries of the farm kid paradox play out differently based on the kinds of family that inhabit a given family farm. But this space betwixt and between is one in which the anthropologist can act as broker, shepherding facts, and concepts between groups who are all experts in their own right. There is a role for the anthropologist in celebrating what is specific about rural childhoods while aiming to ensure that fewer of them end in tragedy.
